# Hybrid Molecules Composed of 2,4-Diamino-1,3,5-triazines and 2-Imino-Coumarins and Coumarins. Synthesis and Cytotoxic Properties

**DOI:** 10.3390/molecules23071616

**Published:** 2018-07-03

**Authors:** Anna Makowska, Franciszek Sączewski, Patrick J. Bednarski, Jarosław Sączewski, Łukasz Balewski

**Affiliations:** 1 Department of Chemical Technology of Drugs, Faculty of Pharmacy, Medical University of Gdańsk, 80-416 Gdańsk, Poland; an.makow@wp.pl (A.M.); lbalewski@gumed.edu.pl (Ł.B.); 2 Department of Pharmaceutical and Medicinal Chemistry, Institute of Pharmacy, University of Greifswald, L.-F.-Jahn Str., D-17489 Greifswald, Germany; bednarsk@uni-greifswald.de; 3 Department of Organic Chemistry, Faculty of Pharmacy, Medical University of Gdańsk, 80-416 Gdańsk, Poland; js@gumed.edu.pl

**Keywords:** 2-imino-2*H*-chromen-3-yl-1,3,5-triazines, 2*H*-chromen-3-yl-1,3,5-triazines, 2,4-diamino-1,3,5-triazines, 2-imino-coumarins, coumarins, hybrid molecules, in vitro anticancer activity

## Abstract

A series of 2-imino-2*H*-chromen-3-yl-1,3,5-triazine compounds **5**–**12**, which are namely hybrids of 2,4-diamino-1,3,5-triazines and 2-imino-coumarins, was synthesized by reacting 2-(4,6-diamine-1,3,5-triazin-2-yl)acetonitriles **1**–**4** with 2-hydroxybenzaldehydes. After this, upon heating in aqueous DMF, 2-imino-2*H*-chromen-3-yl-1,3,5-triazines **10** and **12** were converted into the corresponding 2*H*-chromen-3-yl-1,3,5-triazines **13** and **14**, which are essentially hybrids of 2,4-diamino-1,3,5-triazines and coumarins. The in vitro anticancer activity of the newly prepared compounds was evaluated against five human cancer cell lines: DAN-G, A-427, LCLC-103H, SISO and RT-4. The greatest cytotoxic activity displayed 4-[7-(diethylamino)-2-imino-2*H*-chromen-3-yl]-6-(4-phenylpiperazin-1-yl)-1,3,5-triazin-2-amine (11, IC_50_ in the range of 1.51–2.60 μM).

## 1. Introduction

It is a well-established fact that numerous medical disorders may be caused by defects in more than one specific biological target, such as a receptor and an enzyme. These disease states cannot be adequately addressed by the classical ‘one target, one molecule’ approach [1,2,3]. A promising strategy to tackle complex multifactorial diseases involves the design of hybrid molecules as a stable chemical combination of two drug molecules acting at different targets [4,5,6,7,8,9,10,11,12,13,14,15,16]. Such “dual-acting compounds” combine two distinct chemical entities.

The 1,3,5-triazine moiety represents an extraordinary fragment in several promising classes of compounds with an interesting pharmacological profile [17,18]. Since the 1,3,5-triazine scaffold is present in anticancer drugs, such as altretamine [19], trimelamol [20] and irsogladine [21], a considerable amount of attention has recently been paid to the 1,3,5-triazine derivatives with anticancer activity [22,23,24,25,26]. Thus, 1,3,5-triazines derivatives were identified as apoptosis inductors [27] due to being potent inhibitors of telomerase activity, which is associated with cell proliferation [28] and microtubule-binding agents [28,29,30]. In addition, 1,3,5-triazines with a well-defined antitumor mechanism of action include inhibitors of VEGF-R2 receptor tyrosine kinase [31], tyrosine kinase Tie-2 (tyrosine kinase with immunoglobulin-like and epidermal growth factor-like domains) [32], cyclin-dependent kinase [33] and protein kinase CK2 [34], whereas sulfonamides incorporating the 1,3,5-triazine moiety may act as effective carbonic anhydrase inhibitors [35,36,37].

The coumarin nucleus is a recurring motif in both natural and synthetic compounds of biological interest [38,39,40,41,42], including antiproliferative and cytotoxic agents [43,44,45]. Thus, novobiocin analogues containing a coumarin scaffold were found to inhibit DNA gyrase in breast cancer cells [46], while a number of coumarin derivatives act as inductors of apoptosis [47,48], protein kinase and mitogen-activated protein kinase inhibitors [49,50]; inhibitors of 17β-hydroxysteroid dehydrogenase with therapeutic potential in the treatment of hormone-dependent cancers [51]; and inhibitors of several cancer-related isoforms of carbonic anhydrase [52,53]. It was further demonstrated that coumarin-containing compounds suppress microtubule dynamics, effectively blocking cell cycle progression and resulting in apoptosis [54,55,56], and inhibit angiogenesis. Thus, they represent suitable candidates for the treatment of solid tumors [57]. It should be noted that antitumor activity also exhibit variously substituted imino-icoumarins [58,59,60,61,62].

**Figure 1:**
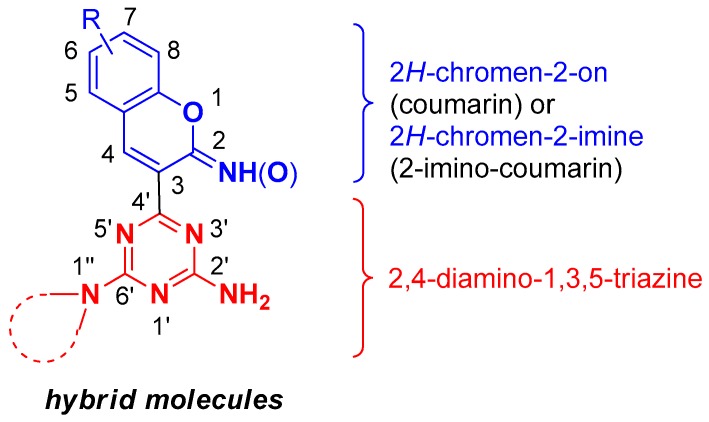
General structure of the hybrid molecules composed of 2,4-diamino-1,3,5-triazines and 2-imino-coumarins or coumarins.

In previous studies, we have synthesized a number of 1,3,5-triazine derivatives with pronounced in vitro antitumor properties [63,64,65,66]. Based on the ideas presented above, we reasoned that compounds incorporating both the 1,3,5-triazine and imino-coumarin pharmacophoric groups could be used in very effective antitumor agents as the hybridization of these two different bioactive molecules, which may lead to a synergistic effect. In this present study, we describe new hybrid compounds synthesized by linking 2,4-diamino-1,3,5-triazinyl moiety with coumarin or 2-imino-coumarin ring system (Figure 1).

## 2. Results and Discussion

### 2.1. Synthesis of 2-Imino-2H-chromen-3-yl-1,3,5-triazine Derivatives (Imino-Coumarin Derivatives)

**Scheme 1:**
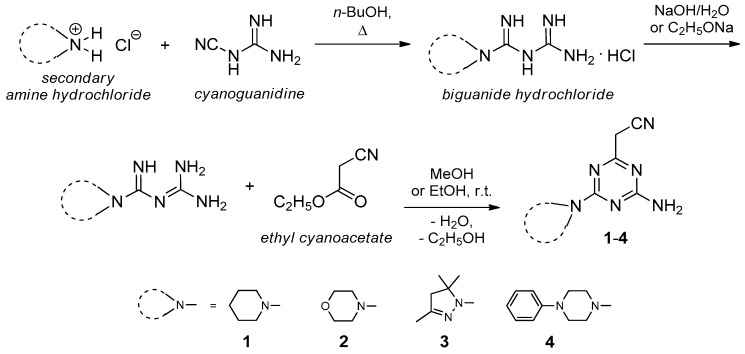
Synthesis of 2-(4,6-diamine-1,3,5-triazin-2-yl)acetonitrile derivatives **1**–**4**.

2,4-Diaminotriazine derivatives **1**–**4** were prepared by cyclocondensation reaction of biguanides [66,67,68] with ethyl cyanoacetate according to the previously described methods [64,66] (Scheme 1).

**Scheme 2:**
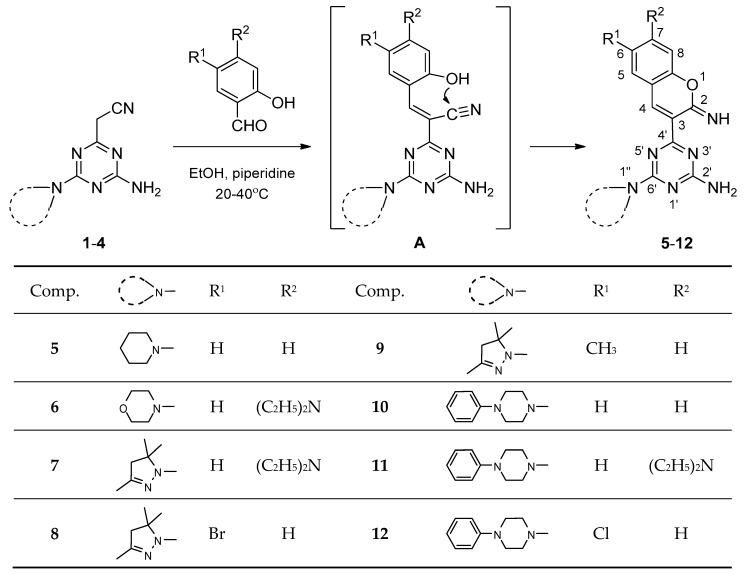
Synthesis of 2-imino-2*H*-chromen-3-yl-1,3,5-triazine derivatives **5**–**12**.

After this, the compounds bearing an active methylene group underwent a reaction with 2-hydroxybenzaldehydes in the presence of piperidine to give the desired hybrid 2-imino-2*H*-chromen-3-yl-1,3,5-triazine compounds **5**–**12** (Scheme 2). The reactions were carried out in 98% ethanol at 20–40 °C in the presence of piperidine as a basic catalyst. The best yields (49–61%) were achieved by using 2-hydroxybenzaldehyde with electron-donating substituent, which was mainly 4-(diethylamino)-2-hydroxybenzaldehyde.

The structures of the 2-imino-coumarin derivatives **5**–**12** were confirmed by elemental analysis, IR and NMR spectra. In the IR spectra, N-H stretching vibrations of the C=N-H group and the primary amine group (NH_2_) of the 1,3,5-triazine ring are observed in a range of 3500 to 3200 cm^−1^. In turn, ^1^H-NMR spectra contain a characteristic singlet representing the proton C4-H of the coumarin ring in a range of 7.43–8.57 ppm, while the proton signal of the imino group C=NH appears in the region of 10.45–11.00 ppm.

However, during spectroscopic characterization of the compounds **7** and **9** containing pyrazoline moiety, we noted the presence of two rotamers with doubled signals found for the protons of the C=NH group in the ^1^H-NMR spectra, which were recorded in DMSO-*d*_6_ solution at 20–22 °C (293.15–295.15 K). The ratio of the rotamers, which was deduced from the integration of the C=NH proton signals, was 1:1 (see experimental section).

**Figure 2:**
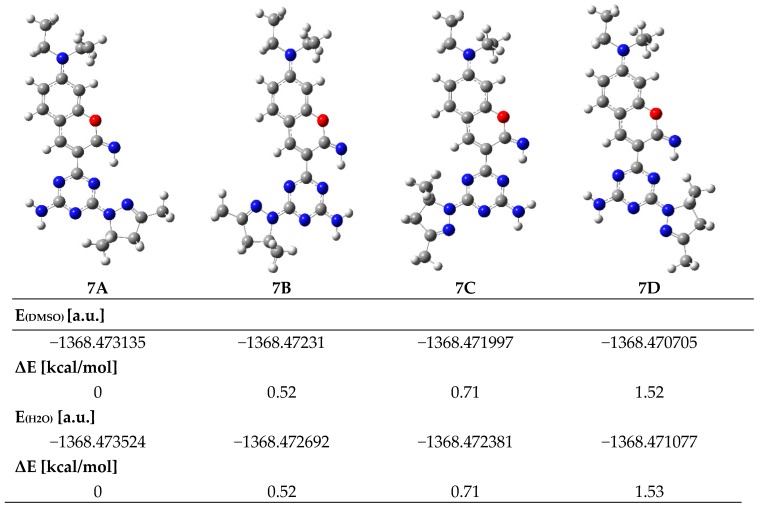
Structures of optimized rotamers **7A**–**D** and corresponding electronic energies (E) and relative energies (ΔE) calculated at B3LYP/6–31(d) level of theory [70] using polarizable continuum model (PCM) solvation model [71].

To obtain a better insight into the origin of signal doubling and the structures of the possible rotamers, we performed quantum chemical calculations for hybrid compound **7** [69]. The four possible rotamers **A**, **B**, **C** and **D** generated from the rotation around the bond axis C3-C4′ (rotation of 2-imino-coumarin) and C6′-N1′′ (rotation pf pyrrazoline) are shown in Figure 2.

**Figure 3:**
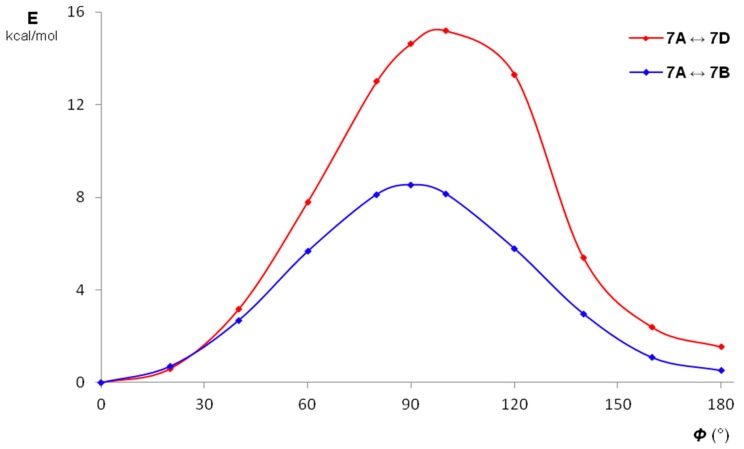
Profiles of the total energy difference with respect to the 180° rotation of single bonds C3–C4′ (**7A**↔**7B**) and C6′–N1′′ (**7A**↔**7D**).

The structure **7A** was proven to be the lowest energy rotameric form both in DMSO and aqueous solution, while the energy differences between **7A** and rotamers **7B**, **7C** and **7D** were very low (0.52–1.53 kcal/mol). Therefore, we determined the barriers of C3-C4′ and C6′-N1′′ bond rotations (Figure 3) and found that the barrier of C6′-N1′′ rotation was much higher (15.2 kcal/mol) than those of C3-C4′ rotation (8.5 kcal/mol). It is significantly easier to overcome the later barrier under normal conditions (14–20 kcal/mol), which suggests that the two separate pairs of rotamers (**7A**, **7B**) and (**7C**, **7D**) may exist in the solution at room temperature, leading to a doubling of the C2=NH proton signal.

### 2.2. Synthesis of 2H-Chromen-3-yl-1,3,5-triazine Derivatives (Coumarin Derivatives)

**Scheme 3:**
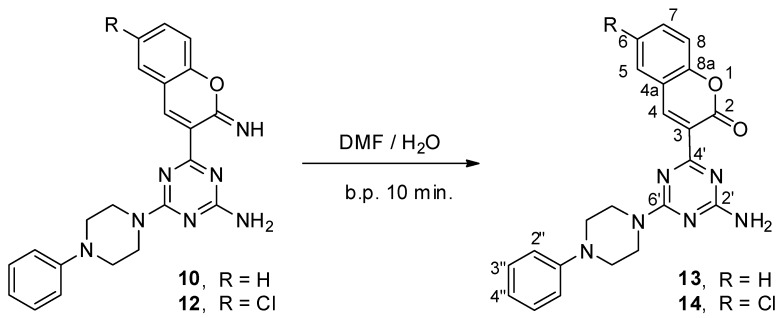
Synthesis of 2*H*-chromen-3-yl-1,3,5-triazine derivatives **13** and **14**.

In a series of experiments aimed at the purification of 2-imino-2*H*-chromen-3-yl-1,3,5-triazines **10** and **12** by means of crystallization, we observed that using dimethylformamide containing 10% of water results in the hydrolysis of the imino group, which results in the formation of coumarin derivatives **13** and **14** (Scheme 3). Thus, the imino-coumarins were proven to be rather unstable under aqueous conditions and the presence of a mineral acid is not required for the hydrolysis of imino-coumarin derivatives as described previously [72].

The structures of the newly prepared compounds **13** and **14** were confirmed by elemental analyses and spectroscopic data (IR and NMR). Thus, in the IR spectra, strong absorptions that are attributable to the carbonyl group (C=O) at 1735 cm^−1^ are observed. In turn, the most diagnostic feature of the ^1^H-NMR spectra is the absence of signals corresponding to the protons of the C2=NH imino group. The characteristic C4-H proton signals of coumarin ring are found at 8.55 ppm (compound **13**) and 8.54 ppm (compound **14**).

The ^13^C-NMR spectrum recorded for 2*H*-chromen-3-yl-1,3,5-triazine derivative 13 (Scheme 3) revealed three signals of C2′=N, C4′=N and C6′=N at 168.95, 167.12 and 164.73 ppm, which confirmed the presence of triazine moiety. The signals of the carbonyl group (C2=O), C8a (quaternary carbon atom) and C4-H of coumarin ring are located at 157.50, 154.13 and 144.41 ppm, respectively. The spectrum of 13 shows a signal at 151.25 ppm, which may be assigned to the quaternary carbon atom of phenylpiperazine. Carbon atoms of phenyl rings (C2′′-H, C3′′-H, C4′′-H of phenylpiperazine and C3, C4a, C5-H, C6-H, C7-H, C8-H of coumarin) give signals in the range of 133.25–116.21 ppm. The aliphatic carbons of piperazine moiety are observed at the highest field (48.62 and 42.78 ppm).

### 2.3. In Vitro Cytotoxic Activity of 2-Imino-2H-chromen-3-yl-1,3,5-triazines (Imino-Coumarins ***5***–***12***) and 2H-Chromen-3-yl-1,3,5-triazines (Coumarins 13, 14)

**Figure 4:**
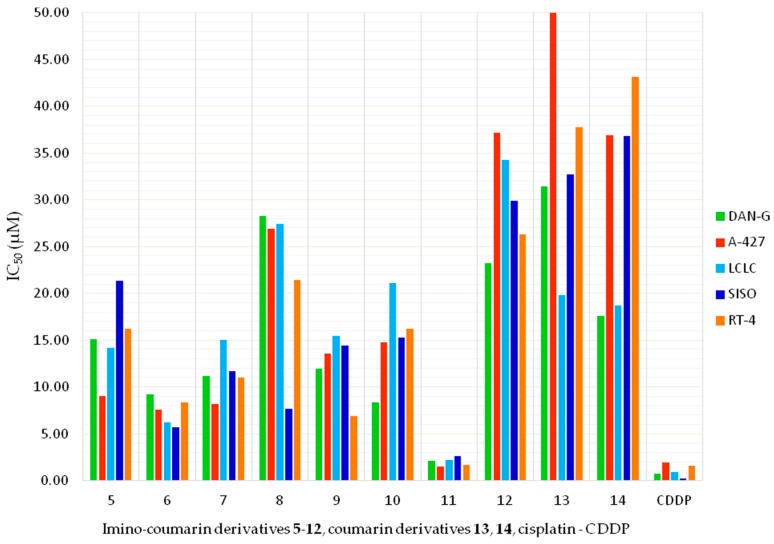
IC_50_ (µM) values of 2-imino-2H-chromen-3-yl-1,3,5-triazines **5**–**12** and 2*H*-chromen-3-yl-1,3,5-triazines **13**, **14** on five human tumor cell lines (DAN-G, A-427, LCLC, SISO, RT-4) compared to cisplatin (CDDP).

The in vitro cytotoxic properties of compounds were evaluated by the crystal violet microtiter plate assay as described earlier [73] with a panel of five human tumor cell lines: human pancreatic cancer cell line DAN-G, human lung cancer line A-427, human non-small cell lung cancer cell line LCLC-103H, human cervix cancer cell line SISO and human urinary bladder cancer cell line RT-4. This assay measures the antiproliferative potencies of compounds towards actively dividing cancer cells. Primary screening was performed to indicate whether a compound possesses enough activity at a concentration of 20 μM to inhibit cell growth by 50%. The secondary screening was aimed at the determination of cytotoxic potencies. The IC_50_ values were obtained after 96-h exposure to the 2-imino-coumarin derivatives **5**–**12** and the coumarin derivatives **13** and **14**. The IC_50_ values calculated from the dose–response data are presented in Table 1 and Figure 4.

The compounds tested can be divided into two series: (1) derivatives **5**–**9** containing a small heterocyclic moiety at the position 6’ of the triazine ring and (2) analogues **10**–**12** substituted with a bulky lipophilic 4-phenylpiperazine moiety. In the first series, the most potent substances were compounds **6** and **7** with the basic electron-donating diethylamino substituent at the position 7 of 2-iminocoumarin ring (IC_50_ in the range of 5.67–9.21 μM and 8.16–15.02 μM, respectively). On the other hand, the lowest activity showed the analogue **8** bearing electron-withdrawing Br substituent at the position 6 (IC_50_ in the range of 7.69–28.25 μM).

The same pattern was seen in the second series of 4-phenylpiperazine-containing compounds. The substitution of **10** with a basic electron-withdrawing Cl substituent, which creates **12**, considerably reduced the cytotoxic activity (IC_50_ in the range of 8.35–21.12 μM vs. 26.32–37.19 μM), while the introduction of a basic electron-donating diethylamino group at the position 7 resulted in the most potent compound **11**, which showed slightly lower cytotoxic activities than cisplatin (IC_50_ values 1.51–2.60 μM vs. 0.06–0.15 mM).

It should be noted that the transformation of 2-iminocoumarins **10** and **12** into the corresponding coumarins **13** and **14** did not improve their cytotoxic properties (see Table 1 and Figure 4).

## 3. Experimental Section

The melting points were determined with a Boëtius apparatus and are uncorrected. The infrared spectra were obtained on KBr pastilles using a Nicolet 380 FT-IR (Thermo Fisher Scientific, Waltham, MA, USA). Magnetic resonance spectra (NMR) were recorded with a Varian Gemini 200 BB (200 MHz) spectrometer (Varian Inc. Palo Alto, CA, USA) and Varian Unity Inova 500 (500 MHz) spectrometer in 200 MHZ in a DMSO-*d*_6_ solution. The residual peak of the solvent was used as an internal standard. Chemical shifts (δ) are given in ppm. Coupling constants (*J*) are given in Hz. The elemental analyses of carbon, hydrogen and nitrogen determined for compounds were within ±0.4% of the theoretical values.

All cell culture reagents were purchased from Sigma (Deisenhofen, FRG). The cancer cell lines used included the following: human large cell lung carcinoma (LCLC-103H), human urinary bladder carcinoma (5637), human lung carcinoma (A-427), human uterine cervical adenocarcinoma (SISO), human bladder cell carcinoma (RT-4) and human pancreas cell adenocarcinoma (DAN-G). These cancer cell lines were obtained from the German Collection of Microorganisms and Cell Cultures (DSMZ, Braunschweig, FRG). The culture medium for cell lines was a RPMI-1640 medium containing 2 g/L HCO_3_ and 10% FCS. Cells were incubated in a humid atmosphere of 5% CO_2_ at 37 °C in 75 cm^2^ plastic culture flasks (Sarstedt, Nümbrecht, FRG) and were passaged shortly before becoming confluent. For the cytotoxicity studies, 100 μL of a cell suspension was seeded into 96-well microtiter plates (Sarstedt) at a density of 1000 cell per well except for the LCLC-103H cell line, which was plated out at 250 cells per well. One day after plating, the cells were treated with the test substance at five concentrations per compound. The 1000-fold concentrated stock solutions in DMF or DMSO were serially diluted by 50% in DMF or DMSO to give the feed solutions, which were diluted by 500-fold in the culture medium. The controls received DMF or DMSO. Each concentration was tested in eight wells, with each well receiving 100 μL of the medium containing the substance. The concentration ranges were chosen to bracket the expected IC_50_ values as best as possible. The cells were incubated for 96 h. After this, the medium was removed and replaced with 1% glutaraldehyde/PBS. Optical density (OD) was measured at λ = 570 nm by use of a Sunrise plate reader (Anthos 2010, Salzburg, Austria). Corrected T/C values were calculated according to the following equation:

(*T*/*C*)_corr(%)_ = (O.D._T_ − O.D._c.0_)/(O.D._C_ − O.D._c.0_) × 100 (1)

where O.D._T_ is the mean absorbance of the treated cells; O.D._C_ is the mean absorbance of the controls; and O.D._c.0_ is the mean absorbance at the time that the drug was added. The IC_50_ values were estimated by a linear least-square regression of the *T/C*_corr_ values compared to the logarithm of the substance concentration. Only the concentrations that yielded *T/C*_corr_ values between 10% and 90% were used in the calculation. The reported IC_50_ values are the averages of three independent experiments.

### 3.1. General Procedure for Preparation of Biguanide Hydrochlorides (Scheme 1)

Cyanoguanidine (dicyandiamide) (2.86 g, 34 mmol) was added to an appropriate solution of amine hydrochloride (34 mmol) in anhydrous *n*-butanol (10 mL). The mixture was carefully heated until the exothermic reaction was initiated (ca. 90 °C) before being stirred at 122–123 °C for 8 h. After cooling, the mixture was stirred for a further 6 h at room temperature. The next day, the precipitate was filtered, washed with *n*-butanol and isopropanol and purified by crystallization using methanol.

Another method described in reference [74] involves the fusion of an equimolar mixture of an amine hydrochloride and cyanoguanidine at 130–150 °C for 0.5–2 h.

### 3.2. Synthesis of 2-[4,6-Diamine-1,3,5-triazin-2-yl]acetonitrile Derivatives ***1***, ***3*** and ***4***

Sodium metal (0.584 g, 25.4 mmol) was added slowly with stirring to anhydrous ethanol (36 mL), before the mixture was stirred under gentle reflux. The appropriate amount of biguanide hydrochloride (25.4 mmol) was added to the obtained solution of sodium ethoxide in ethanol. The mixture was cooled and stirred at room temperature for 2 h. The ethyl cyanoacetate (25.4 mmol, 2.03 mL, d = 1.418 g/mL) was added dropwise at room temperature (20–22 °C) to a stirred solution over the next hour. The precipitate was filtered, washed with ethanol and crystallized from the appropriate solvent.

The compounds **1**, **3** and **4** were obtained by the following procedure:

*2-[4-Amino-6-(piperidin-1-yl)-1,3,5-triazin-2-yl]acetonitrile* (**1**), Yield 2.6 g (47%); m.p. 126–127 °C (MeOH); IR (KBr) ν_max_ (cm^−1^): 3462, 3355, 3245, 3006, 2961, 2927, 2860, 2257, 1651, 1578, 1550, 1515, 1465, 1446, 1389, 1369, 1290, 994, 805; ^1^H-NMR (200 MHz, DMSO-*d*_6_) δ (ppm): 1.40–1.70 (m, 6H, 3xCH_2_), 3.60–3.80 (m, 4H, 2xCH_2_), 3.85 (s, 2H, CH_2_); 6.97 (br.s, 2H, NH_2_). Anal. calcd for C_10_H_14_N_6_ (218.26): C, 55.03; H, 6.47; N, 38.50. Found: C, 54.98; H, 6.38; N, 38.46.

*2-[4-Amino-6-(3,5,5-trimethyl-4,5-dihydro-1H-pyrazol-1-yl)-1,3,5-triazin-2-yl]acetonitrile* (**3**), Yield 3.00 g (48%); m.p. 244–245 °C (*n*-BuOH), reference [64]: 246–247 °C; IR (KBr) ν_max_ (cm^−1^): 3354, 3214, 2951, 2924, 2261, 1655, 1539, 1469, 1447, 1380, 1335, 1149, 969, 932, 807, 608. ^1^H-NMR (200 MHz, DMSO-*d*_6_) δ (ppm): 1.57 (s, 6H, 2xCH_3_), 1.99 (s, 3H, CH_3_), 2.83 (s, 2H, CH_2_), 3.92 (s, 2H, CH_2_); 7.10 (s, 1H, NH), 7.40 (s, 1H, NH). Anal. calcd for C_11_H_15_N_7_ (245.28): C, 53.86; H, 6.16; N, 39.97. Found: C, 53.78; H, 6.18; N, 39.86.

*2-[4-Amino-6-(4-phenylpiperazin-1-yl)-1,3,5-triazin-2-yl]acetonitrile* (**4**), Yield 4.5 g (60%); m.p. 207–209 °C (DMF-H_2_O), reference [66]: 205–208 °C; IR (KBr) ν_max_ (cm^−1^): 3433, 3322, 3149, 2963, 2908, 2814, 2259, 1656, 1553, 1494, 1476, 1443, 1386, 1370, 1332, 1282, 1240, 1146, 1004, 919, 801, 761, 696; ^1^H-NMR (500 MHz, DMSO-*d*_6_) δ (ppm): 3.14–3.28 (m, 4H, 2xCH_2_), 3.82–3.90 (m, 4H, 2xCH_2_), 3.91 (s, 2H, CH_2_), 6.82 (t, 1H, CH), 6.99 (d, *J* = 7.8 Hz, 2H, 2xCH), 7.06 (s, 1H, NH), 7.15 (s, 1H, NH), 7.24 (t, 2H, 2xCH). Anal. calcd for C_15_H_17_N_7_ (295.34): C, 61.00; H, 5.80; N, 33.20. Found: C, 60.92; H, 5.74; N, 33.12.

### 3.3. Synthesis of 2-(4-amino-6-morpholino-1,3,5-triazin-2-yl)acetonitrile (***2***)

Ethyl cyanoacetate (3.46 g, 3.44 mL, 30.57 mmol, d = 1.418 g/mL) was added dropwise to a solution of *N*-carbamimidoylmorpholine-4-carboximidamide (5.23 g, 30.57 mmol) in anhydrous methanol (48 mL) over 1 h. The mixture was stirred for 2 h at room temperature (20–22 °C). The precipitate was filtered and washed with cold methanol (5 °C). The product was crystallized from methanol. Yield 3.2 g (48%); m.p. 164–165 °C (MeOH), reference [66]: 178–180 °C; IR (KBr) ν_max_ (cm^−1^): 3352, 3315, 3202, 2974, 2951, 2258, 1650, 1574, 1530, 1471, 1447, 1380, 1335, 1152, 969, 808, 631; ^1^H-NMR (200 MHz, DMSO-*d*_6_) δ (ppm): 3.55–3.63 (m, 4H, 2xCH_2_), 3.64–3.75 (m, 4H, 2xCH_2_); 3.88 (s, 2H, CH_2_), 7.04 (s, 1H, NH), 7.12 (s, 1H, NH). Anal. calcd. for C_9_H_12_N_6_O (220.23): C, 49.08; H, 5.49; N, 38.16. Found: C, 49.02; H, 5.41; N, 38.18.

### 3.4. Synthesis of 2-Imino-2H-chromen-3-yl-1,3,5-triazine Derivatives ***5***–***12*** (General Procedure)

An appropriate amount of 2-hydroxybenzaldehyde derivative (13 mmol) was added gradually to a suspension of an appropriate amount of 1,3,5-triazineacetonitrile 1–4 (10 mmol) in 98% ethanol (30 mL). After 3 min of stirring, piperidine (0.2 mL) was added dropwise to the solution. The mixture was heated for 30 min at 40 °C and cooled. Stirring was continued at room temperature (20–22 °C) for 18 h. The precipitate was filtered, washed with anhydrous ethanol (3 × 2 mL) and dried. The imino-coumarin derivatives 5–12 were proven to be unstable upon heating in protic solvents. They also decomposed when we attempted chromatographic purification on silica gel. Therefore, the products washed with cold ethanol were used for structural and biological investigations.

The following compounds were obtained by the following procedure:

*4-(2-Imino-2H-chromen-3-yl)-6-(piperidin-1-yl)-1,3,5-triazin-2-amine* (**5**), Yellow powder 0.67 g (21%); m.p. 216–217 °C; IR (KBr) ν_max_ (cm^−1^): 3339, 3213, 3047, 2941, 2850, 1650, 1613, 1598, 1541, 1513, 1456, 1395, 1294, 1237, 818, 760; ^1^H-NMR (200 MHz, DMSO-*d*_6_) δ (ppm): 1.40–1.75 (m, 6H, 3xCH_2_), 3.65–3.85 (m, 4H, 2xCH_2_), 6.95–7.30 (m, 4H, 2xCH + NH_2_), 7.45–7.60 (m, 1H, CH), 7.67 (d, *J* = 7.3 Hz, 1H, CH), 8.50 (s, 1H, CH), 10.71 (s, 1H, NH). Anal. calcd for C_17_H_18_N_6_O (322.36): C, 63.34; H, 5.63; N, 26.07. Found: C, 63.28; H, 5.51; N, 25.98.

*4-[7-(Diethylamino)-2-imino-2H-chromen-3-yl]-6-morpholino-1,3,5-triazin-2-amine* (**6**), Yellow powder, yield 2.41 g (61%); m.p. 232–233 °C; IR (KBr) ν_max_ (cm^−1^): 3443, 3333, 3210, 2967, 2924, 2854, 1654, 1603, 1508, 1440, 1398, 1351, 1280, 1236, 1137, 816; ^1^H-NMR (500 MHz, DMSO-*d*_6_) δ (ppm): 1.14 (t, 6H, 2xCH_3_), 3.44 (q, 4H, 2xCH_2_), 3.60–3.69 (m, 4H, 2xCH_2_), 3.70–3.85 (m, 4H, 2xCH_2_), 6.39 (s, 1H, CH), 6.55 (d, *J* = 8.8 Hz, 1H, CH), 7.00 (s, 1H, NH), 7.12 (s, 1H, NH), 7.39 (d, *J* = 8.8 Hz, 1H, CH), 8.45 (s, 1H, CH), 10.48 (s, 1H, NH). Anal. calcd for C_20_H_25_N_7_O_2_ (395.46): C, 60.74; H, 6.37; N, 24.79. Found: C, 60.68; H, 6.31; N, 24.57.

*4-[7-(Diethylamino)-2-imino-2H-chromen-3-yl]-6-(3,5,5-trimethyl-4,5-dihydro-1H-pyrazol-1-yl)-1,3,5-triazin-2-amine* (**7**), Orange powder, yield 2.05 g (49%); m.p. 232–233 °C; IR (KBr) ν_max_ (cm^−1^): 3464, 3289, 3175, 2974, 2930, 1652, 1603, 1439, 1383, 1352, 1332, 1248, 1143, 1075, 811; ^1^H-NMR (500 MHz, DMSO-*d*_6_) δ (ppm): 1.11 (t, 6H, 2xCH_3_), 1.61 (s, 6H, 2xCH_3_), 1.99 (s, 3H, CH_3_), 2.84 (s, 2H, CH_2_), 3.42 (q, 4H, 2xCH_2_), 6.37 (s, 1H, CH), 6.51 (dd, *J*_1_ = 2.4 Hz, *J*_2_ = 8.8 Hz, 1H, CH), 7.00 (br.s, 2H, NH_2_), 7.33 (d, *J* = 8.8 Hz, 1H, CH), 8.32 (s, 1H, CH), 10.45 (br.s, 0.5H, NH), 10.82 (br.s, 0.5H, NH). Anal. calcd for C_22_H_28_N_8_O (420.51): C, 62.84; H, 6.71; N, 26.65. Found: C, 62.68; H, 6.64; N, 26.58.

*4-(6-Bromo-2-imino-2H-chromen-3-yl)-6-(3,5,5-trimethyl-4,5-dihydro-1H-pyrazol-1-yl)-1,3,5-triazin-2-amine* (**8**), Orange powder, yield 1.62 g (38%); m.p. 207–208 °C; IR (KBr) ν_max_ (cm^−1^): 3208, 2923, 2855, 1672, 1605, 1533, 1439, 1376, 1330, 1261, 1237, 1155, 813; ^1^H-NMR (200 MHz, DMSO-*d*_6_) δ (ppm): 1.53 (s, 6H, 2xCH_3_), 1.61 (s, 3H, CH_3_), 3.76 (s, 2H, CH_2_), 7.13 (br.s, 2H, NH_2_), 7.18 (d, *J* = 8.8 Hz, 1H, CH), 7.66 (dd, *J*_1_ = 2.3 Hz, *J*_2_ = 8.8 Hz, 1H, CH), 7.98 (d, *J* = 2.3 Hz, 1H, CH), 8.50 (s, 1H, CH), 10.82 (s, 1H, NH). Anal. calcd for C_18_H_18_BrN_7_O (428.29): C, 50.48; H, 4.24; N, 22.89. Found: C, 50.42; H, 4.12; N, 22.84.

*4-(2-Imino-6-methyl-2H-chromen-3-yl)-6-(3,5,5-trimethyl-4,5-dihydro-1H-pyrazol-1-yl)-1,3,5-triazin-2-amine* (**9**), Yellow powder, yield 1.12 g (31%); m.p. 172–173 °C; IR (KBr) ν_max_ (cm^−1^): 3448, 3209, 1649, 1522, 1458, 1399, 1379, 1339, 810, 608; ^1^H-NMR (500 MHz, DMSO-*d*_6_) δ (ppm): 1.64 (s, 6H, 2xCH_3_), 2.02 (s, 3H, CH_3_), 2.34 (s, 3H, CH_3_), 2.89 (s, 2H, CH_2_), 7.12 (d, *J* = 8.2 Hz, 1H, CH), 7.24 (br.s, 2H, NH_2_), 7.36 (d, *J* = 8.2 Hz, 1H, CH), 7.43 (s, 1H, CH), 8.36 (br.s, 0.5H, CH), 8.40 (br.s, 0.5H, CH); 10.63 (br.s, 0.5H, NH), 11.00 (br.s, 0.5H, NH). Anal. calcd for C_19_H_21_N_7_O (363.42): C, 62.79; H, 5.82; N, 26.98. Found: C, 62.68; H, 5.78; N, 26.92.

*4-(2-Imino-2H-chromen-3-yl)-6-(4-phenylpiperazin-1-yl)-1,3,5-triazin-2-amine* (**10**), Yellow powder, yield 1.07 g (27%); m.p. 193–194 °C; IR (KBr) ν_max_ (cm^−1^): 3475, 3295, 3178, 3061, 2851, 1655, 1615, 1600, 1526, 1444, 1398, 1375, 1286, 1233, 1040, 1007, 938, 814, 754; ^1^H-NMR (200 MHz, DMSO-*d*_6_) δ (ppm): 3.15–3.30 (m, 4H, 2xCH_2_), 3.85–4.05 (m, 4H, 2xCH_2_), 6.82 (t, 1H, CH), 7.00 (d, *J* = 8.1 Hz, 2H, 2xCH), 7.15–7.40 (m, 6H, 4xCH + NH_2_), 7.50–7.60 (m, 1H, CH), 7.68 (d, *J* = 7.4 Hz, 1H, CH), 8.57 (s, 1H, CH), 10.72 (s, 1H, NH); ^13^C NMR (50 MHz, DMSO-*d*_6_) δ (ppm): 41.06 (2 overlapping signals), 48.59 (2 overlapping signals), 115.46, 116.21 (2 overlapping signals), 118.76, 119.58, 121.38, 123.62, 129.26 (2 overlapping signals), 129.54, 133.22, 137.91, 151.20, 153.85, 159.18, 164.10, 166.31, 167.94. Anal. calcd for C_22_H_21_N_7_O (399.45): C, 66.15; H, 5.30; N, 24.55. Found: C, 66.09; H, 5.26; N, 24.48.

*4-[7-(Diethylamino)-2-imino-2H-chromen-3-yl]-6-(4-phenylpiperazin-1-yl)-1,3,5-triazin-2-amine* (**11**), Orange powder, yield 2.54 g (54%); m.p. 158–159 °C; IR (KBr) ν_max_ (cm^−1^): 3496, 3392, 3315, 3252, 3204, 3054, 2971, 2853, 1652, 1604, 1510, 1439, 1397, 1350, 1288, 1235, 1160, 1137, 1007, 815, 756, 690; ^1^H-NMR (500 MHz, DMSO-*d*_6_) δ (ppm): 1.14 (t, 6H, 2xCH_3_), 3.20–3.25 (m, 4H, 2xCH_2_), 3.44 (q, 4H, 2xCH_2_), 3.85–4.00 (m, 4H, 2xCH_2_), 6.41 (s, 1H, CH), 6.55 (d, *J* = 8.8 Hz, 1H, CH), 6.83 (t, 1H, CH), 6.95–7.05 (m, 3H, 2xCH + NH), 7.13 (br.s, 1H, NH), 7.25 (t, 2H, 2xCH), 7.40 (d, *J* = 8.8 Hz, 1H, CH), 8.46 (s, 1H, CH), 10.49 (s, 1H, NH). Anal. calcd for C_26_H_30_N_8_O (470.57): C, 66.36; H, 6.43; N, 23.81. Found: C, 66.28; H, 6.41; N, 23.78.

*4-(6-Chloro-2-imino-2H-chromen-3-yl)-6-(4-phenylpiperazin-1-yl)-1,3,5-triazin-2-amine* (**12**), Dark yellow powder, yield 1.43 g (33%); m.p. 196–197 °C; IR (KBr) ν_max_ (cm^−1^): 3480, 3389, 3289, 3195, 3058, 2908, 2855, 1653, 1596, 1521, 1440, 1394, 1290, 1230, 1008, 938, 813, 758, 691; ^1^H-NMR (500 MHz, DMSO-*d*_6_) δ (ppm): 3.18–3.28 (m, 4H, 2xCH_2_), 3.85–4.05 (m, 4H, 2xCH_2_), 6.80–6.85 (m, 1H, CH), 7.02 (d, *J* = 7.8 Hz, 2H, 2xCH), 7.20–7.30 (m, 4H, 3xCH + NH), 7.34 (br.s, 1H, NH), 7.57 (dd, *J*_1_ = 2.0 Hz, *J*_2_ = 8.8 Hz, 1H, CH), 7.86 (s, 1H, CH), 8.56 (s, 1H, CH), 10.81 (s, 1H, NH). Anal. calcd for C_22_H_20_ClN_7_O (433.89): C, 60.90; H, 4.65; N, 22.60. Found: C, 60.82; H, 4.62; N, 22.51.

### 3.5. Synthesis of 2H-chromen-3-yl-1,3,5-triazine Derivatives ***13*** and ***14*** (General Procedure)

An appropriate amount of 2-imino-2*H*-chromen-3-yl-1,3,5-triazine derivative 10 or 12 (1.5 mmol) was dissolved in DMF containing 10% water (2–4 mL). The mixture was slowly heated with stirring to boiling for 10 min, before being cooled to ambient temperature (20–22 °C). Stirring was continued at room temperature (20–22 °C) and crushed ice was added until a precipitate was formed. The product was filtered, washed with water, dried and crystallized from DMF.

*3-[4-Amino-6-(4-phenylpiperazin-1-yl)-1,3,5-triazin-2-yl]-2H-chromen-2-one* (**13**), Light yellow powder, yield 0.46 g (77%); m.p. 266–267 °C; IR (KBr) ν_max_ (cm^−1^): 3464, 3325, 3165, 3067, 2974, 2833, 1735, 1663, 1628, 1609, 1557, 1524, 1504, 1473, 1442, 1375, 1291, 1232, 979, 816, 749; ^1^H-NMR (500 MHz, DMSO-*d*_6_) δ (ppm): 3.16–3.24 (m, 4H, 2xCH_2_), 3.85–3.99 (m, 4H, 2xCH_2_), 6.83 (t, 1H, CH), 7.01 (d, *J* = 8.3 Hz, 2H, 2xCH), 7.06 (br.s, 1H, NH), 7.13 (br.s, 1H, NH), 7.25 (t, 2H, 2xCH), 7.41 (t, 1H, CH), 7.46 (d, *J* = 8.3 Hz, 1H, CH), 7.70 (t, 1H, CH), 7.88 (d, *J* = 6.8 Hz, 1H, CH), 8.55 (s, 1H, CH); ^13^C-NMR (50 MHz, DMSO-d_6_) δ (ppm): 42.78 (2 overlapping signals), 48.62 (2 overlapping signals), 116.21 (2 overlapping signals), 116.27, 118.76, 119.57, 124.93, 125.56, 129.25 (2 overlapping signals), 129.68, 133.25, 144.41, 151.25, 154.13, 157.50, 164.73, 167.12, 168.95. Anal. calcd for C_22_H_20_N_6_O_2_ (400.43): C, 65.99; H, 5.03; N, 20.99. Found: C, 65.79; H, 4.92; N, 20.91.

*3-[4-Amino-6-(4-phenylpiperazin-1-yl)-1,3,5-triazin-2-yl]-6-chloro-2H-chromen-2-one* (**14**), Yellow powder, yield 0.48 g (74%); m.p. 245–246 °C; IR (KBr) ν_max_ (cm^−1^): 3482, 3314, 3220, 2988, 2948, 2908, 2850, 1735, 1645, 1595, 1569, 1555, 1529, 1471, 1442, 1373, 1288, 1233, 1045, 946, 827, 814; ^1^H-NMR (200 MHz, DMSO-*d*_6_) δ (ppm): 3.05–3.45 (m, 4H, 2xCH_2_), 3.75–4.05 (m, 4H, 2xCH_2_), 6.81 (t, 1H, CH), 6.90–7.14 (m, 3H, 2xCH + NH), 7.15–7.30 (m, 2H, CH + NH), 7.35–7.55 (m, 2H, 2xCH), 7.67 (t, 1H, CH), 7.87 (d, *J* = 7.0 Hz, 1H, CH), 8.54 (s, 1H, CH). Anal. calcd for C_22_H_19_ClN_6_O_2_ (434.88): C, 60.76; H, 4.40; N, 19.32. Found: C, 60.68; H, 4.36; N, 19.28.

## 4. Conclusions

The results of the biological studies indicate that hybrid compounds **5**–**14** have rather weak cytotoxic properties. However, significant antiproliferative potency is associated with a diethylamino substituent at the position 7 of the coumarin ring (compounds **6** and **11**). Importantly, compound **11** with a bulky 4-phenylpiperazine moiety installed at the position 6’ of the triazine ring showed similar potency to cisplatin against several of the cell lines. It is too early to speculate on the mechanism of action of the compound **11**. However, it is well known that coumarins are minor groove binders and exhibit the intercalative mode of binding properties with DNA [75,76]. Therefore, the presence of diethylamino group may increase the efficiency of the intercalative binding due to the extra non-covalent force between the substituent and DNA grooves. The most potent 2,4-diamino-1,3,5-triazine-imino-coumarin **11** may serve as a lead structure for further development of new antitumor drugs. Thus, the hybrid molecule composed of 2,4-diamino-1,3,5-triazine and 2-iminocoumarin was proven to be a promising heterocyclic scaffold for the construction of novel cytotoxic compounds. The syntheses of analogues containing lipophilic substituents at the position 6′ of the triazine ring and electron-donating substituents at the positions 5, 6, 7 or 8 of the imino-coumarin moiety, along with quantitative structure-activity relationship (QSAR) studies, are planned.

## Figures and Tables

**Table 1:**
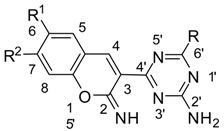
Cytotoxic activity of 2-imino-2*H*-chromen-3-yl-1,3,5-triazines **5**–**12** and 2*H*-chromen-3-yl-1,3,5-triazines **13**, **14** on five human tumor cell lines (IC_50_ ± SD, µM) compared to cisplatin (CDDP).

No.	R	R^1^	R^2^	X	DAN-G	A-427	LCLC	SISO	RT-4
5	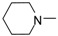	H	H	NH	15.12 ± 5.79	9.04 ± 4.55	14.17 ± 12.98	21.33 ± 2.69	16.24 ± 6.15
6	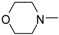	H	(C_2_H_5_)_2_N	NH	9.21 ± 0.59	7.62 ± 0.27	6.18 ± 1.26	5.67 ± 0.80	8.37 ± 1.66
7	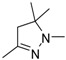	H	(C_2_H_5_)_2_N	NH	11.19 ± 0.55	8.16 ± 3.08	15.02 ± 0.82	11.64 ± 1.69	11.04 ± 6.49
8	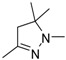	Br	H	NH	28.25 ± 4.06	26.87 ± 2.45	27.42 ± 4.00	7.69 ± 1.72	21.40 ± 2.39
9	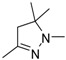	CH_3_	H	NH	11.91 ± 0.52	13.56 ± 1.45	15.47 ± 1.84	14.44 ± 2.49	6.91 ± 0.62
10	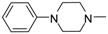	H	H	NH	8.35 ± 0.87	14.79 ± 0.45	21.12 ± 2.69	15.24 ± 0.73	16.24 ± 6.15
11	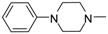	H	(C_2_H_5_)_2_N	NH	2.14 ± 0.25	1.51 ± 0.17	2.21 ± 0.39	2.60 ± 0.37	1.66 ± 0.25
12	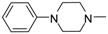	Cl	H	NH	23.26 ± 6.62	37.19 ± 6.09	34.24 ± 3.05	29.86 ± 6.13	26.32 ± 2.99
13	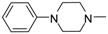	H	H	O	31.44 ± 5.98	>50	19.82 ± 1.12	32.73 ± 2.33	37.73 ± 3.13
14	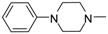	Cl	H	O	15.87 ± 1.73	25.95 ± 10.95	17.15 ± 1.56	29.15 ± 7.64	36.14 ± 7.00
cisplatin (CDDP) reference [73]					0.73 ± 0.34	1.96 ± 0.54	0.90 ± 0.19	0.24 ± 0.06	1.61 ± 0.16
